# BLProt: prediction of bioluminescent proteins based on support vector machine and relieff feature selection

**DOI:** 10.1186/1471-2105-12-345

**Published:** 2011-08-17

**Authors:** Krishna Kumar Kandaswamy, Ganesan Pugalenthi, Mehrnaz Khodam Hazrati, Kai-Uwe Kalies, Thomas Martinetz

**Affiliations:** 1 Institute for Neuro- and Bioinformatics, University of Lübeck, 23538 Lübeck, Germany; 2 Graduate School for Computing in Medicine and Life Sciences, University of Lübeck, 23538 Lübeck, Germany; 3 Bioinformatics Group, Bioscience Core Lab, King Abdullah University of Science and Technology (KAUST), Kingdom of Saudi Arabia; 4 Institute for Signal Processing, University of Lübeck, 23538 Lübeck, Germany; 5 Centre for Structural and Cell Biology in Medicine, Institute of Biology, University of Lübeck, Germany

## Abstract

**Background:**

Bioluminescence is a process in which light is emitted by a living organism. Most creatures that emit light are sea creatures, but some insects, plants, fungi etc, also emit light. The biotechnological application of bioluminescence has become routine and is considered essential for many medical and general technological advances. Identification of bioluminescent proteins is more challenging due to their poor similarity in sequence. So far, no specific method has been reported to identify bioluminescent proteins from primary sequence.

**Results:**

In this paper, we propose a novel predictive method that uses a Support Vector Machine (SVM) and physicochemical properties to predict bioluminescent proteins. BLProt was trained using a dataset consisting of 300 bioluminescent proteins and 300 non-bioluminescent proteins, and evaluated by an independent set of 141 bioluminescent proteins and 18202 non-bioluminescent proteins. To identify the most prominent features, we carried out feature selection with three different filter approaches, ReliefF, infogain, and mRMR. We selected five different feature subsets by decreasing the number of features, and the performance of each feature subset was evaluated.

**Conclusion:**

BLProt achieves 80% accuracy from training (5 fold cross-validations) and 80.06% accuracy from testing. The performance of BLProt was compared with BLAST and HMM. High prediction accuracy and successful prediction of hypothetical proteins suggests that BLProt can be a useful approach to identify bioluminescent proteins from sequence information, irrespective of their sequence similarity. The BLProt software is available at http://www.inb.uni-luebeck.de/tools-demos/bioluminescent%20protein/BLProt

## Background

Bioluminescence is an enchanting process in which light is produced by a chemical reaction within an organism [1,2]. Bioluminescence is found in various organisms like ctenophora, bacteria, certain annelids, fungi, fish, insects, algae, squid, etc. Most of these organisms are found in marine, freshwater, and terrestrial habitats [2-4]. The bioluminescence mechanism involves two chemicals, namely luciferin, a substrate, and the enzyme luciferase [1,5]. Luciferase catalyses the oxidation of luciferin, resulting in light and an intermediate called oxyluciferin. Sometimes, the luciferin catalyzing protein (the equivalent of a luciferase) and a co-factor such as oxygen are bound together to form a single unit called photoprotein. This molecule is triggered to produce light when a particular type of ion is added to the system. The proportionality of the light emission makes a clear distinction between a photoprotein and a luciferase [5]. Photoproteins are capable of emitting light in proportion to the amount of the catalyzing protein, but in luciferase-catalyzed reactions, the amount of light emitted is proportional to the concentration of the substrate luciferins [2].

Different creatures produce different colors of light, from violet through red [3,6]. The different colors of light produced are often dependent on the roles the light plays, the organism in which it is produced, and the varieties of chemicals produced. The dominant color on land is green, because it reflects best against green plants. The most common bioluminescent color in the ocean is blue. This color transmits best through sea water, which can scatter or absorb light.

Bioluminescence serves a variety of functions, but many of them are still unknown. The known functions include camouflage, finding food, attraction of prey, attraction of mates, repulsion by way of confusion, signaling other members of their species, confusing potential predators, communication between bioluminescent bacteria (quorum sensing), illumination of prey, burglar alarm, etc [3-5].

The application of bioluminescence promises great possibilities for medical and commercial advances. Bioluminescent proteins serve as invaluable biochemical tools with applications in a variety of fields including gene expression analysis, drug discovery, the study of protein dynamics and mapping signal transduction pathways, bioluminescent imaging, toxicity determination, DNA sequencing studies, estimating metal ions such as calcium, etc [7-14].

The detailed analysis of bioluminescence proteins helps to understand many of the functions which are still unknown and also helps to design new medical and commercial applications. Due to advances in sequencing technologies, huge amount of data is available in various databases [15]. Despite tremendous progress in the annotation of protein, there are no existing online tools available for the prediction of bioluminescent proteins using primary protein sequences.

A Support Vector Machine (SVM) is a supervised learning algorithm, which has been found to be useful in the recognition and discrimination of hidden patterns in complex datasets [16]. SVM has been successfully applied in various fields of computational biology, e.g., protein sequence/structure analysis, micro-array and gene expression analysis [16-18].

In this work, we present a novel prediction method that uses a Support Vector Machine (SVM) and physicochemical properties to predict bioluminescent proteins. So far, bioinformatics and statistical learning methods like Support Vector Machine and Random Forest have not been explored for the prediction of bioluminescent proteins. In this paper, we report a SVM approach to identify bioluminescent proteins from sequence information, irrespective of the sequence similarity.

## Results and Discussion

### Performance of similarity based search using PSI-BLAST

Similarity search methods play a vital role in the classification of proteins. PSI-BLAST is the most popular similarity based search method for searching sequence databases [19]. PSI-BLAST search for each query sequence was performed against the database of 441 bioluminescent proteins that were used for the training and testing. Three iterations of PSI-BLAST were carried out at a cut-off *E*-value of 0.001. It was observed that 280 bioluminescent proteins showed similarity (BLAST hit) with other bioluminescent protein sequences (evalue-0.001). The performance of the sequence similarity method drops when there is no significant sequence similarity between two proteins. Hence, such an alignment-based method would rarely yield satisfactory predictions. Therefore, there is a need for alignment-free methods (machine learning models) for predicting bioluminescent proteins.

### Prediction of bioluminescent proteins by BLProt

**Figure 1:**
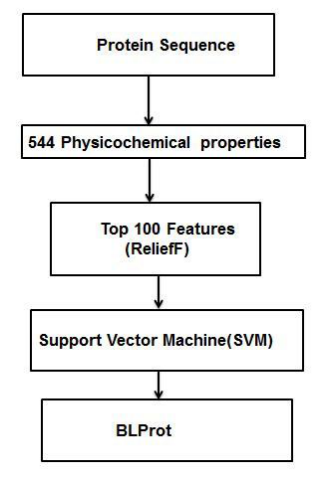
**Workflow of BLProt**.

A SVM classifier was applied to predict bioluminescent proteins. Each sequence was encoded by 554 features. The detailed flowchart of our work is shown in Figure 1. The model was trained with a dataset containing 300 bioluminescent protein sequences and 300 non-bioluminescent protein sequences. BLProt achieved 75.16% training accuracy (5 fold cross-validations) with all of the 544 physicochemical properties (Table 1).

**Table 1:** Performance of the SVM using different feature subsets selected by ReliefF

Feature subset	Sensitivity (%)	Specificity (%)	MCC	Test Accuracy (%)	CV Accuracy
75 features	69.50	77.13	0.4663	73.86	77.16
100 features	74.47	84.21	0.5904	80.06	80.00
200 features	68.09	81.58	0.5022	75.83	78.00
300 features	67.38	82.11	0.5017	75.83	78.67
400 features	64.54	86.32	0.5260	77.04	78.00
500 features	65.96	85.79	0.5323	77.34	78.00
All features	63.12	78.19	0.4182	71.73	75.16

MCC - Matthew's correlation coefficient, CV-Cross validation

To identify the most prominent features, we carried out feature selection with three different filter approaches, ReliefF, Info Gain, and mRMR. We selected five different feature subsets by decreasing the number of features, and the performance of each feature subset was evaluated (see Table 1, Table 2, and Table 3). The best performance of 80% training accuracy was achieved with ReliefF selecting 100 features. Hence, this is chosen as the final model for our work.

**Table 2:** Performance of the SVM using different feature subsets selected by Info Gain

Feature subset	Sensitivity (%)	Specificity (%)	MCC	Test Accuracy (%)	CV Accuracy
100 features	69.50	74.21	0.4351	72.21	74.83
200 features	76.60	75.79	0.5193	76.13	78.00
300 features	70.92	77.37	0.4821	74.62	78.33
400 features	68.09	77.89	0.4611	73.72	78.17
500 features	68.09	84.21	0.5326	77.34	78.33
All features	63.12	78.19	0.4182	71.73	75.16

MCC - Matthew's correlation coefficient, CV-Cross validation

**Table 3:** Performance of the SVM using different feature subsets selected by mRMR

Feature subset	Sensitivity (%)	Specificity (%)	MCC	Test Accuracy (%)	CV Accuracy
100 features	65.96	84.21	0.5134	76.44	78.33
200 features	65.25	84.74	0.5132	76.44	78.5
300 features	65.96	83.68	0.5072	76.13	78.5
400 features	65.96	83.68	0.5072	76.13	78.33
500 features	65.96	83.68	0.5072	76.13	78.5
All features	63.12	78.19	0.4182	71.73	75.16

MCC - Matthew's correlation coefficient, CV-Cross validation

After training, we tested our algorithm on the test dataset consisting of 141 bioluminescent protein sequences and 18202 non-bioluminescent proteins sequences. The maximum accuracy of 80.06% with 74.47% sensitivity and 84.21% specificity was obtained using the top 100 features (ReliefF, Table 1).

**Figure 2:**
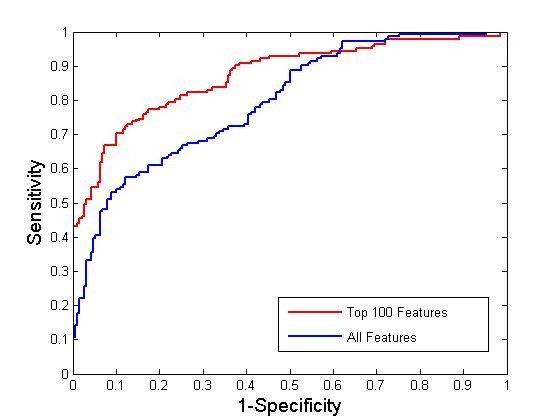
**ROC Plot for SVM models using all and the top 100 features (ReliefF)**.

Figure 2 presents a chart with the true positive rates and false positive rates on the test data at different thresholds for the classifiers using all the features and the top 100 features, respectively (ReliefF). The area under curve for all features was 0.79 and for the top 100 features was 0.87, respectively.

### Comparison of BLProt with HMM and BLAST

The performance of BLProt was compared with other sequence search methods, namely HMM and PSI-BLAST using 141 bioluminescent proteins [19,20]. PSI-BLAST search for each sequence was carried out against the Swissprot database with an E value of 0.1. HMM search for each query sequence was performed against the HMM profile obtained from the Pfam database (Pfam release 23) [21]. Out of 141 proteins, 114 proteins were correctly predicted by BLProt. PSI-BLAST and HMM correctly predicted 99 and 76 proteins, respectively.

Our algorithm was further evaluated by 9 hypothetical proteins obtained from the INTERPRO, CDD and KEGG databases [22-24] (Table 4). Our approach correctly predicted all proteins as bioluminescent proteins. The performance of our algorithm was compared with PSI-BLAST and HMM [19,20]. Out of these 9 proteins, the PSI-BLAST search retrieved bioluminescent protein hits from the Swissprot database for only 4 proteins. No hits were found for the remaining 5 proteins. Similarly, HMM search against the Pfam database returned no hits for 3 proteins. This result indicates that BL-Prot is a useful approach for predicting bioluminescent proteins from sequence information in the absence of sequence similarity.

**Table 4:** Prediction result for 9 potential Bioluminescent proteins

GI	BLProt	PSI-BLAST	HMM	Source of annotation
156529049	BLP	Non-BLP	BLP	INTERPRO
37528019	BLP	BLP	Non-BLP	KEGG
37528018	BLP	BLP	BLP	CDD
45440453	BLP	Non-BLP	BLP	INTERPRO
45440453	BLP	Non-BLP	BLP	INTERPRO
153796564	BLP	Non-BLP	Non-BLP	INTERPRO
49257059	BLP	BLP	BLP	CDD
159576911	BLP	BLP	Non-BLP	CDD
49257059	BLP	Non-BLP	BLP	INTERPRO

BLP - Bioluminescent protein; Non-BLP - Non-bioluminescent protein

CDD - Conserved Domain Database

### Comparison with other machine learning methods

The proposed SVM model was compared with several state-of-the-art classifiers such as J4.8, PART, Random Forest, Adaboost and IBK [25-29]. We compared the performance of BLProt with the other approaches using the same feature subset (top 100 features from ReliefF). All models were tested on the test dataset containing 141 positive and 18202 negative sequences. The results are shown in Table 5. The prediction accuracy of BLProt is about 7% and 12% higher than that of J4.8 and PART, respectively. Although the sensitivity of BLProt, Random Forest and IBK is comparable, BLProt is superior in specificity and concerning the MCC values.

**Table 5:** Comparison of BLProt with other machine learning methods

Method	Sensitivity (%)	Specificity (%)	MCC	Accuracy (%)
J4.8	69.50	75.79	0.4518	73.11
PART	63.12	72.11	0.3519	68.28
IBK	76.60	69.47	0.4556	72.51
Random Forest	75.18	73.16	0.4787	74.02
AdaBoost	68.79	72.63	0.4117	71.00
BLProt	74.47	84.21	0.5904	80.06

MCC - Matthew's correlation coefficient

## Conclusion

Bioluminescence, the chemically-induced production of light within a living organism, is a process which varies among organisms. The application of bioluminescent proteins has wide medical and commercial values, which explains the interest for the identification of novel bioluminescent proteins. In this study, we developed a method for predicting bioluminescent proteins from its primary sequence using ReliefF coupled with SVM. BLProt will help the experimental biologist to predict bioluminescence from a protein sequence and, thus, help to avoid unnecessary experiments. The BLProt program and dataset is available at http://www.inb.uni-luebeck.de/tools-demos/bioluminescent%20protein/BLProt

## Methods

### Dataset

We obtained 300 bioluminescent proteins from seed proteins of the Pfam database [21]. To enrich the dataset, we performed PSI-BLAST search against non-redundant sequence database with stringent threshold (E-value 0.01) [19]. Redundant sequences that have >=40% sequence similarity were removed from the dataset using CD-HIT [30]. After careful manual examination, a total of 441 bioluminescent proteins were selected for the positive dataset.

#### Training set

300 bioluminescent proteins were selected from 441 bioluminescent proteins for the positive dataset. 300 non-bioluminescent proteins for the negative set were randomly taken from seed proteins of Pfam protein families, which are unrelated to bioluminescent proteins.

#### Test set

The remaining 141 bioluminescent proteins served as a positive dataset for testing. The negative dataset was created from the seed proteins of non-bioluminescent proteins, which are selected from seed proteins of non-bioluminescent protein Pfam protein families [21]. The negative sequences present in the training dataset were removed. Furthermore, non-bioluminescent protein domains with less than 40 amino acids were excluded from the negative set. Finally, the test dataset consisted of 141 bioluminescent proteins and 18202 non-bioluminescent proteins.

### The steps of the Algorithm

The following steps were performed and are described in detail below:

1. Get the protein sequence data from the Pfam database.

2. Assign class labels: bioluminescent proteins = +1 (positive class); non-bioluminescent proteins = -1 (negative class).

3. Convert all the sequences to numerical equivalents based on physicochemical properties

4. Get the top 100 features from ReliefF feature selection algorthim.

5. Partition the data into training and test sets.

6. Run the SVM classifier on the training set.

7. Run the SVM classifier on the test set to assess the generalization.

### Representation of the proteins as vectors of physicochemical properties

The first step is the conversion of the protein sequence into a vector of physicochemical properties. For this purpose we used the AAindex. The AAindex is a database of numerical indices representing various physicochemical and biochemical properties of amino acids and pairs of amino acids [31]. This database provides a collection of 544 such physicochemical properties. We converted each protein sequence into a 544 dimensional feature vector. Each physicochemical property value is determined by the sum of the physicochemical property values of all residues of the sequence, divided by the length of the sequence.

### Support vector machines for classification

The Support Vector Machine (SVM) has been successfully used to solve various problems in bioinformatics [16-18,32-34]. The SVM is a supervised machine learning method which is based on statistical learning theory [35]. When used as a binary classifier, the SVM constructs a hyperplane in a kernel feature space that acts as the decision surface between the two classes. The SVM maximizes the margin of separation between the hyperplane and those data points nearest to it. The details of the formulation and solution methodology of the SVM for binary classification tasks can be found in [35,36]. Only relevant details are provided here.

Let *x_i _*∈ *R^n^*, *i *= 1, 2,...,*n *be training instances and *y_i _*∈ {-1, +1} be their corresponding target class labels.

Given a new instance x, the decision on its class affiliation can be made depending upon the sign of the function

(1) $$f\left(x\right)=\overset{m}{\underset{i=\text{1}}{\sum }}y_{i}\alpha_{i}K\left(x_{i},x\right)+b$$

where *m *is the number of input instances having non-zero values of the Lagrange multipliers (α*_i_*)(usually a subset of *n *known as the support vectors). The α_i _are obtained by solving a quadratic optimization problem on the training instances. *b *is the bias term.

*K*(*x_i_, x*) denotes the kernel function. In the present study, we used the Gaussian kernel, defined by

(2) $$K\left(x_{i},x_{j}\right)=\text{exp}\left(-\gamma {∥x_{i}-x_{j}∥}^{\text{2}}\right)$$

where *γ *defines the width of the kernel.

All computations were performed using the LIBSVM - 2.81 standard package [37]. The user-defined parameters, i.e. the kernel parameter *γ *and the regularization parameter C (allows one to trade off training error vs. model complexity), were optimized by a grid search. A small value for C corresponds to a small model complexity and guarantees a small deviation of the test error from the training error. However, the training error might be large. A large C provides small training errors, however, with an increase of the model complexity the probability that the test error is significantly worse than the training error increases. A C going to infinity leads to a hard-margin SVM [38]. The grid search was done using a 5-fold cross-validation. For that purpose the dataset was randomly divided into 5 subsets. The training and validation was carried out 5 times for each model using one distinct set for validation and the remaining four for training. The performance of the model, as reported in Table 1, is the average performance over these 5 sets.

### Feature Selection

In this work, the main purpose of conducting feature selection is to remove possible redundant features from the original feature set. By redundant, we mean that the feature has negligible influence on the final classification performance. To identify the prominent features that separate the positive and negative class, we used Info Gain, ReliefF and minimal-redundancy-maximal-relevance (mRMR) algorithms.

In Info Gain, we calculate the information gain for each feature, and rank them according to this measure, which indicates the gain of information [39]. Different models were built for the top 100, 200, 300, 400 and 500 features. In ReliefF, we choose the features that can be most distinguished between classes. It evaluates the worth of a feature by repeatedly sampling an instance and considering the value of the given feature for the nearest instance of the same and different class [40]. Again, different models were built for the top 100, 200, 300, 400 and 500 features.

The minimal-redundancy-maximal-relevance (mRMR) algorithm is a sequential forward selection algorithm first developed by Peng et al. to analyze the importance of different features [41]. mRMR uses the mutual information to select M features that best fulfill the minimal redundancy and maximal relevance criterion. A detailed description of the mRMR method can be found in (Peng et al., 2005). Different models were built for the top 100, 200, 300, 400 and 500 features.

### Performance Evaluation of the SVM

For the purpose of assessing the generalization capability, we calculated the accuracy, sensitivity, specificity and Matthew's Correlation Coefficients (MCC) on an independent test set:

(3) $$\text{Accuracy}=\frac{\left(TP+TN\right)}{\left(TP+FP+TN+FN\right)}$$

(4) $$\text{Sensitivity}=\frac{TP}{\left(TP+FN\right)}$$

(5) $$\text{Specificity}=\frac{TN}{\left(TN+FP\right)}$$

(6) $$MCC=\frac{TP*TN-FP*FN}{\sqrt{\left(TP+FP\right)\left(TN+FN\right)\left(TP+FN\right)\left(TN+FP\right)}}$$

where TP - True Positive, FP - False Positive, TN - True Negative and FN - False Negative.

The Matthew's correlation coefficient ranges from -1 ≤ MCC ≤ 1. A value of MCC = 1 indicates the best possible prediction while MCC = -1 indicates the worst possible prediction (or anti-correlation). MCC = 0 would be expected for a random prediction scheme.

## Competing interests

The authors declare that they have no competing interests.

## Authors' contributions

KKK, GP and MKH contributed equally to the analysis and manuscript preparation. KUK and TM coordinated the study, helped drafting the manuscript, and critically revised its content. All authors read and approved the manuscript.
